# Associations between the keratinized mucosa width and the underlying alveolar bone dimensions at partial edentulous molar sites: a retrospective cross-sectional study

**DOI:** 10.1186/s12903-024-04590-2

**Published:** 2024-07-15

**Authors:** Ziyao Han, Cui Wang, Yiping Wei, Gang Yang, Wenjie Hu, Kwok-Hung Chung

**Affiliations:** 1 Department of Periodontology, National Center for Stomatology & National Clinical Research Center for Oral Diseases & National Engineering Research Center of Oral Biomaterials and Digital Medical Devices, Beijing Key Laboratory of Digital Stomatology, Peking University School and Hospital of Stomatology, Beijing, China; 2 NHC Research Center of Engineering and Technology for Computerized Dentistry, Beijing, China; 3 Department of Restorative Dentistry, University of Washington, Seattle, WA USA

**Keywords:** Alveolar process atrophy, Alveolar ridge, Buccal mucosa, Cone-beam computed tomography

## Abstract

**Background:**

The assessment of hard and soft tissue at edentulous sites is important for subsequent implant treatment design. The aim of the present study was to explore the associations between the keratinized mucosa width (KMW) and the underlying alveolar bone dimensions at partial edentulous molar sites.

**Methods:**

In this retrospective study, a total of 110 patients with at least one missing molar were selected. The buccal KMW of the edentulous molar sites was evaluated. Cone-beam computed tomography scans were collected, and the height discrepancy between the alveolar crest and the buccal bone plate (H_C−B_) as well as the alveolar bone height (ABH) were measured. The KMW was compared among the H_C−B_ and ABH groups at both maxillary and mandibular sites. Linear regression and generalized estimation equations (GEEs) were used to explore the associations between the KMW and alveolar bone dimensions at α = 0.05.

**Results:**

Among the 110 patients, 158 edentulous molar sites were analyzed. The average H_C−B_ and ABH were significantly lower at the maxillary sites (1.26 ± 1.62 mm, 11.62 ± 3.94 mm) than at the mandibular sites (3.67 ± 2.85 mm, 14.91 ± 3.01 mm, *p* < 0.001). The KMW was significantly lower at sites with H_C−B_ > 2 mm than at sites with H_C−B_ ≤ 2 mm both in the maxilla and mandible (*p* < 0.001). No significant differences were found between the KMW at sites with ABH < 10 mm and sites with ABH ≥ 10 mm (*p* > 0.05). Linear regression and GEEs analyses revealed that the H_C−B_ was significantly associated with the KMW (*B* = -0.339, *p* < 0.001), while the association between the KMW and the ABH was not statistically significant (*B* = -0.046, *p* = 0.352).

**Conclusions:**

The buccal KMW at edentulous molar sites was significantly associated with the H_C−B_. Alveolar ridges presenting with a sloped configuration were more prone to possess a narrower band of keratinized mucosa. Both hard and soft tissue augmentation should be considered for implant treatment at these sites. The correlations of dynamic changes between the KMW and alveolar bone dimensions after tooth extraction should be further investigated.

## Background

As one of the components that comprises the peri-implant phenotype, an adequate keratinized mucosa width (KMW) is beneficial for peri-implant health [1, 2]. Thorough plaque removal is difficult for implants surrounded only by alveolar mucosa, which could lead to a greater risk of developing peri-implant disease [3, 4]. Soft tissue deficiencies that occur before implant placement are mainly due to postextraction tissue remodeling, and a diminished KMW is usually discovered at lower molar sites [5, 6]. For edentulous sites designed to receive implant placement after hard and soft tissue remodeling are mostly completed, a narrow KMW before implant placement is often related to peri-implant KMW deficiency after loading, which could increase the risk of peri-implant diseases [7–9]. Thus, it is necessary for clinicians to evaluate the peri-implant KMW before implant placement and determine whether soft tissue augmentation procedures should be performed [10].

Alveolar ridge remodeling is a physiological phenomenon that occurs after tooth extraction [11]. Changes in soft tissue dimensions following tooth extraction correlate with underlying alveolar bone resorption [12, 13]. During the initial 6-month period following tooth extraction, vertical and horizontal bone resorption reach 11-22% and 29-63%, respectively [12–17]. Previous reports indicate that a limited reduction in the vertical ridge dimension occurs following single-tooth extraction, while horizontal resorption reaches 50% [11, 12]. Alveolar ridge atrophy at postextraction sites is more pronounced on the facio-coronal aspect [18]. The buccal bone plate, which mainly consists of bundle bone, is a tooth-dependent structure and undergoes more significant resorption than the lingual plate following tooth loss [11, 19]. This difference results in the coronal margin of the alveolar crest shifting more lingually, especially at molar extraction sites [11, 20, 21]. The different resorption patterns previously observed in the buccal and lingual plates of the edentulous ridge often result in the following: (1) a sloping configuration of the alveolar bone, (2) a height discrepancy between the alveolar crest and the buccal bone plate, and (3) coronal shifting of the mucogingival junction (MGJ) at edentulous sites [22, 23]. Thus, shrinkage of the buccal keratinized mucosa accompanied by postextraction alveolar ridge atrophy is commonly observed in clinical practice, most notably in edentulous molar regions [5].

Postextraction soft tissue thickness and hard tissue dimensions have been reported in previous studies [13, 24, 25]. The characteristics of the periodontal phenotype, such as the bone phenotype, could also play a role in spontaneous postextraction healing dynamics [18]. Sites with a thin facial bone phenotype will present more pronounced hard and soft tissue dimensional changes [26]. Fewer changes in soft tissue thickness or supracrestal soft tissue height after tooth extraction are observed at sites with thicker facial bone [27]. However, scientific literature providing evidence to support the association between the KMW and the underlying hard tissue at edentulous sites is scarce. The aim of the present study was to explore the associations between the KMW and the underlying alveolar bone dimensions at edentulous molar sites.

## Methods

### Study population and inclusion and exclusion criteria

The present study was designed as a retrospective cross-sectional study. The study was registered in the Chinese Clinical Trial Registry (ChiCTR-2,200,064,979) on October 25th, 2022 and reported following the STROBE guidelines. Patients with missing molars admitted to the Department of Periodontology, Peking University School and Hospital of Stomatology for implant reconstruction between June 2013 and October 2020 were screened as suitable subjects. The ethics approval of the study was obtained from the Institutional Review Board of Peking University School and Hospital of Stomatology (No. PKUSSIRB-202,058,143) in December 2020 and was in accordance with the Declaration of Helsinki revised in 2013. All participants signed an informed consent before participating in the study, and the anonymized data was extracted. The inclusion and exclusion criteria were as follows:

The inclusion criteria were as follows:

- older than 18 years,
- at least one molar lost (excluding third molars) more than 6 months prior,
- periodontally healthy according to the consensus report by Chapple et al. [28],
- and available cone-beam computed tomography (CBCT) images of the edentulous molar sites.

The exclusion criteria were as follows:

- molars extracted within 6 months;
- poorly controlled systemic diseases that could affect wound healing;
- history of soft tissue augmentation;
- history of alveolar ridge preservation, sinus floor elevation or any other bone augmentation procedures;
- a medication history that affects soft tissue healing or hyperplasia;
- a history of bisphosphonate use or head and neck radiotherapy.

### Clinical measurements

**Fig. 1:**
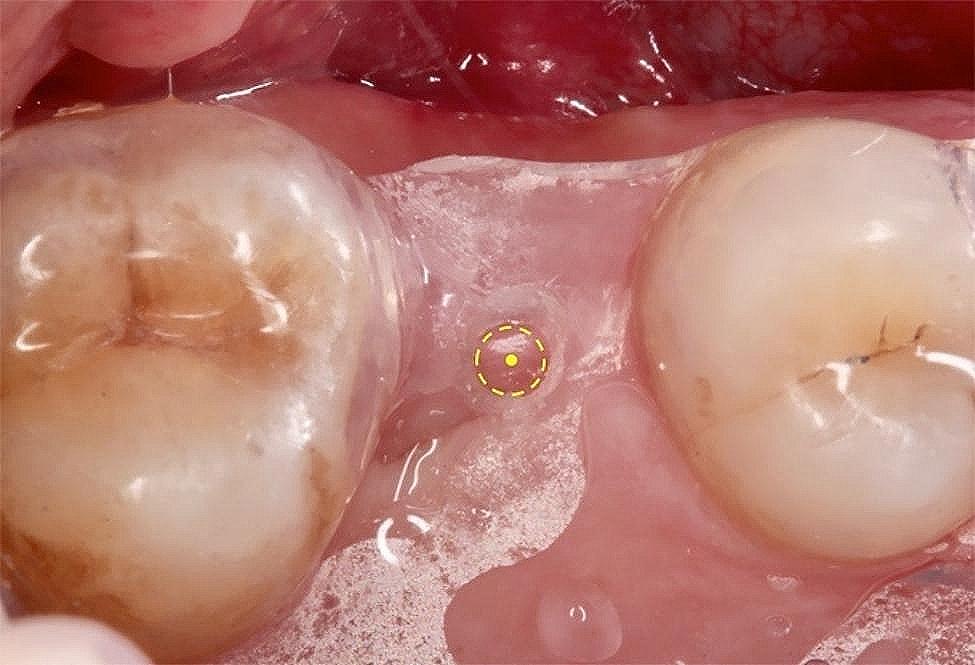
The center of implant placement (the yellow dot) was identified as the reference point with the acrylic stent in place for the measurement of keratinized mucosa width

One experienced examiner completed all the clinical measurements (WH). An acrylic surgical stent indicating the location of implantation was designed for every subject for subsequent implant treatment (Fig. 1), and the Williams periodontal probe was used to punch and mark the center of the implant placement (reference point) with the stent in place. The KMW at the midbuccal aspect of the edentulous site was measured from the MGJ to the reference point on the alveolar crest with the Williams periodontal probe, and the measurements were rounded to the nearest 1 mm. The MGJ was identified by the difference in mobility between the keratinized mucosa and alveolar mucosa by pushing the alveolar mucosa coronally.

### Radiographical measurements

**Fig. 2:**
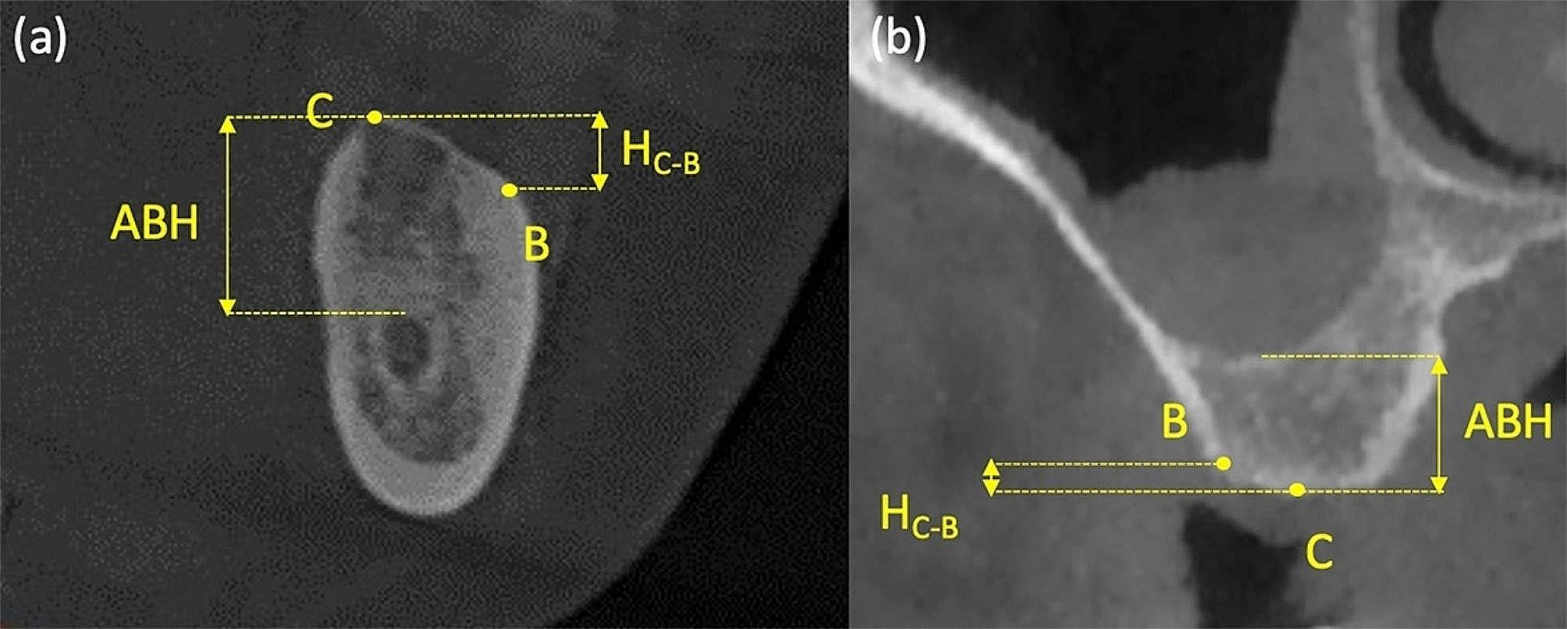
Radiographic measurements on CBCT images. Slices of (**a**) mandibular and (**b**) maxillary edentulous molar sites. C: alveolar crest; B: most coronal point of the buccal bone plate; H_C−B_: the height discrepancy between the alveolar crest and the buccal bone plate; ABH: alveolar bone height

All CBCT scans of the edentulous sites were taken with a 0.2 mm slice thickness, 8 × 8 cm field of view, 360 degrees, and voxel size of 0.15 mm with the same imaging unit (NewTom VG, Aperio Services, Italy). The exposure parameters were as follows: 15 s, 110 KVP, and 12–17 mAs. Radiographic measurements were performed on the CBCT images using MIMICS (Materialise, Leuven, Belgium). Slices of the edentulous sites were extracted perpendicular to the alveolar ridge through the center of implant placement, according to Nunes et al. and Braut et al. [29, 30]. The height discrepancy between the alveolar crest and the buccal bone plate (H_C−B_) was measured (Fig. 2). When the most coronal point of the alveolar crest was at the buccal bone plate, the H_C−B_ was recorded as 0 mm. The alveolar bone height (ABH) was measured from the alveolar crest to the superior border of the mandibular canal (mandibular sites, Fig. 2a) or the level of the sinus floor (maxillary sites, Fig. 2b).

### Sample size calculation

The sample size was calculated according to our previous study, with an alpha error of 0.05 and a beta error of 0.1 in a bilateral contrast (1 − *β* = 0.90) [31, 32]. The minimum sample size was calculated to be 115. To increase the strength of the results, all subjects who fulfilled the inclusion criteria were initially included.

### Intra- and interexaminer agreement

All clinical measurements were performed by an experienced periodontist (WH). To test the intra-examiner reliability, 20 edentulous molar sites of the subjects were randomly selected, and the KMWs were measured twice over 2 weeks. The intraclass correlation coefficient (ICC) was 0.983 (95% CI [0.971, 0.995], *p* < 0.001). The CBCT measurements were completed by another two examiners (ZH & CW). All the CBCT images were measured twice within 2 weeks, and the interexaminer intraclass correlation coefficient (ICC) was 0.986 (95% CI [0.979, 0.991], *p* < 0.001).

### Statistical analysis

The dataset was analyzed by SPSS 26.0 (IBM Corporation). All continuous variables are presented as the mean, standard deviation (SD), median and range. The repeated measurements of H_C−B_ and ABH (twice from two examiners) at each site were taken as average values for comparison and correlation analysis. According to Schiegnitz et al. [23] and Nunes et al. [29], the cutoff values of H_C−B_ and ABH were 2 mm and 10 mm, respectively, and the KMW of edentulous sites was compared between groups divided by H_C−B_ and ABH in two arches using the Mann‒Whitney *U* test. Linear regression and generalized estimation equations (GEEs) were used to explore the associations between the KMW and alveolar bone dimensions at edentulous molar sites. The significance was defined as *α* = 0.05.

## Results

### Dataset

After the preliminary screening, 117 individuals with 168 edentulous sites were selected. Five individuals with 6 molar sites were excluded due to incomplete medical records, and 2 individuals with 4 molar sites were excluded because they were receiving bone augmentation procedures before implant placement. A total of 110 individuals (71 males and 39 females, mean age of 51.2 years) with 158 edentulous molar sites were included in the current study. Each patient had 1–3 edentulous molar sites. The sample included 54 edentulous sites in the maxilla and 104 edentulous sites in the mandible (Table 1). The reason for tooth loss was periodontal at 73 sites, and the remaining 85 sites experienced tooth loss for nonperiodontal reasons (Table 1). No statistically significant differences were detected between the KMWs of sites with periodontal and nonperiodontal reasons for tooth loss (3.93 ± 1.99 mm vs. 3.81 ± 2.32 mm, *p* = 0.730).

**Table 1:** Characteristics of patients and the edentulous molar sites

Variable	*N* (%)
**Age (year)**	
Mean ± SD	51.8 ± 9.1
Median (range)	53 (27–73)
**Gender** (*n* [%])	
Male	71 (64.5)
Female	39 (35.5)
**Location** (*n* [%])	
Upper 6	43 (27.2)
Upper 7	11 (7.0)
Lower 6	67 (42.4)
Lower 7	37 (23.4)
**Reasons of tooth loss** (*n* [%])	
Periodontal Non-periodontal	73 (46.2) 85 (53.8)

SD, standard deviation; *N*, sample size

### KMW at edentulous molar sites with different alveolar bone dimensions

Table 2 presents the KMW, H_C−B_ and ABH of the edentulous molar sites at the two arches. In the maxilla, both the average H_C−B_ and ABH were significantly lower than those in the mandibular sites (*p* < 0.001). As illustrated in Table 3, a significant difference (1.72 mm, 95% CI [1.09, 2.35]) was detected between the KMW of edentulous molar sites with H_C−B_ ≤ 2 mm and those with H_C−B_ > 2 mm (4.81 ± 2.12 mm vs. 3.08 ± 1.89 mm, *p* < 0.001). The KMW of the edentulous ridge with H_C−B_ ≤ 2 mm was significantly greater than that of the edentulous ridge with H_C−B_ >2 mm in both the maxilla and mandible (*p* < 0.05, Table 4). The differences (0.34 mm, 95% CI [-0.56, 1.23]) between the KMW of edentulous molar sites with ≥ 10 mm or < 10 mm ABH were not statistically significant (3.81 ± 2.15 mm vs. 4.14 ± 2.27 mm, *p* = 0.460). Sites with ≥ 10 mm ABH had greater KMW when evaluated in two arches, but the differences were not statistically significant (*p* > 0.05).

**Table 2:** The keratinized mucosa width and alveolar bone dimensions (in mm) at edentulous molar sites of different jaws

	Maxilla (mean ± SD)	Mandible (mean ± SD)	*p* value
KMW	5.09 ± 2.19	3.23 ± 1.87	< 0.001*
H_C−B_	1.26 ± 1.62	3.67 ± 2.85	< 0.001*
ABH	11.62 ± 3.94	14.91 ± 3.01	< 0.001*

SD: standard deviation; KMW: keratinized mucosa width; H_C−B_: height discrepancy from the alveolar crest to the buccal bone plate; ABH: alveolar bone height

*Statistically significant difference (*p* < 0.05) using Mann-Whitney *U* test

**Table 3:** Keratinized mucosa width (in mm) at edentulous molar sites with different alveolar bone dimensions

Variable	*N*	KMW (mm)	
		Mean ± SD	Median (range)
**H**_**C−B**_ **(mm)**			
≤ 2	72	4.81 ± 2.12	5 (0–10)
> 2	86	3.08 ± 1.89	3 (0–8)
*p* value		< 0.001*	
Mean differences (95% CI)		1.72 (1.09, 2.35)	
**ABH(mm)**			
< 10	28	4.14 ± 2.27	4 (0–9)
≥ 10	130	3.81 ± 2.15	4 (0–10)
*p* value		0.460	
Mean differences (95% CI)		0.34 (-0.56, 1.23)	

N: sample size; KMW: keratinized mucosa width; H_C−B_: height discrepancy from the alveolar crest to the buccal bone plate; ABH: alveolar bone height; CI: confidence interval

*Statistically significant difference (*p* < 0.05) using Mann-Whitney *U* test

**Table 4:** Keratinized mucosa width (in mm) at edentulous molar sites with different alveolar ridge dimensions

Variable	Maxilla	Mandible
**H**_**C−B**_ **(mm)**		
≤ 2	5.43 ± 2.13	4.16 ± 1.74
> 2	3.92 ± 2.07	2.82 ± 1.79
*p* value	0.034*	0.001*
**ABH(mm)**		
< 10	4.77 ± 2.02	1.83 ± 1.60
≥ 10	5.31 ± 2.31	3.32 ± 1.86
*p* value	0.332	0.063

H_C−B_: height discrepancy from the alveolar crest to the buccal bone plate; ABH: alveolar bone height

*Statistically significant difference (*p* < 0.05) using Mann-Whitney *U* test

### Associations between the KMW and alveolar bone dimensions

**Fig. 3:**
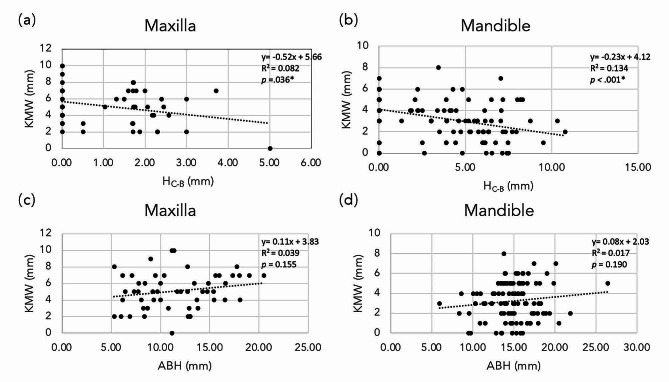
Linear regression plots representing the relationship between the keratinized mucosa width and alveolar ridge dimensions at the (**a**) (**c**) maxillary and (**b**) (**d**) mandibular edentulous molar sites. KMW: keratinized mucosa width; H_C−B_: height discrepancy from the alveolar crest to the buccal bone plate; ABH: alveolar bone height; *p < 0.05

**Fig. 4:**
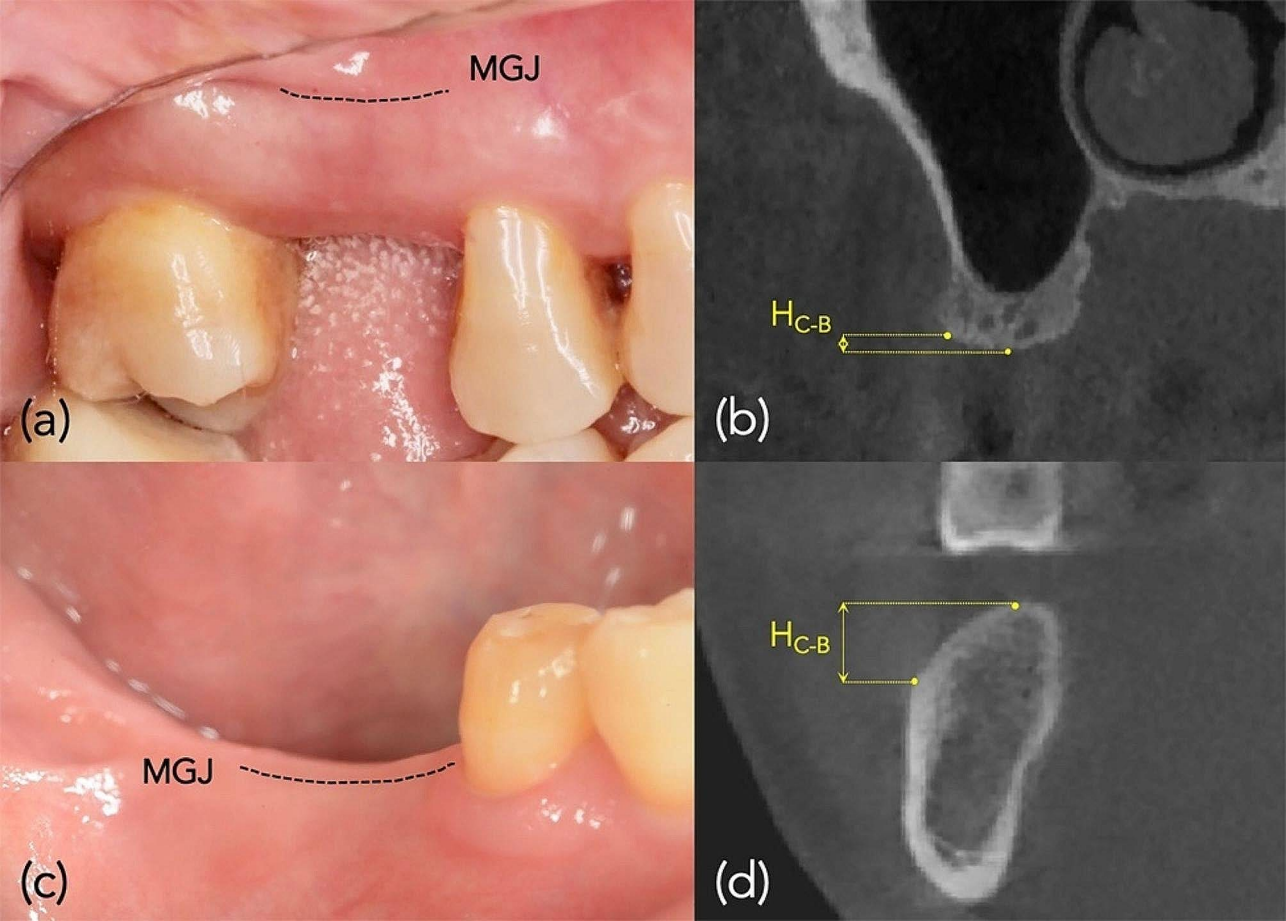
Associations between keratinized mucosa width (KMW) and the height discrepancy between the alveolar crest and the buccal bone plate (H_C−B_) at edentulous molar sites. The mucogingival junction (MGJ) was marked with a black dotted line. (**a**) An edentulous molar site with a wide KMW and (**b**) an underlying bone crest with a low H_C−B_; (**c**) an edentulous molar site with a narrow KMW and (**d**) an underlying bone crest with a high H_C−B_

Linear regression analysis indicated significant negative associations between the KMW and H_C−B_ at both maxillary and mandibular edentulous molar sites (*r* = -0.52, *p* = 0.036; *r* = -0.25, *p* < 0.001) (Fig. 3a, b). No significant associations were found between the KMW and ABH (*p* > 0.05, Fig. 3c, d). As illustrated in Table 5, after adjusting for confounding factors, the GEE analysis revealed a significant association between the KMW and H_C−B_ at edentulous molar sites (*B* = -0.339, *p* < 0.001), while no significant correlations were found between the KMW and ABH (*B* = -0.046, *p* = 0.352). The typical clinical cases demonstrating edentulous molar sites of narrow keratinized mucosa with a high H_C−B_ and wide keratinized mucosa with a low H_C−B_ are presented in Fig. 4.

**Table 5:** Generalized estimation equations analysis of keratinized mucosa width and the associated alveolar bone dimensions

Variables	Wald^2^	B	*p* value
H_C−B_	27.264	-0.339	< 0.001*
ABH	0.867	-0.046	0.352

H_C−B_: height discrepancy from the alveolar crest to the buccal bone plate; ABH: alveolar bone height

*p < 0.05

## Discussion

Hard and soft tissue changes following tooth loss have been widely studied and discussed [11, 13]. However, to our knowledge, the characteristics of the KMW at postextraction edentulous sites and its relationship with underlying hard tissue dimensions remain unclear. This study was conducted by analyzing the edentulous molar sites in a 3-dimensional view with clinical and radiographic measurements. When limiting our scope to edentulous molar sites that have healed without any interventions after tooth loss, the present study revealed that the KMW at an edentulous site was associated with the height discrepancy between the alveolar bone crest and the buccal bone plate.

The height discrepancy between the alveolar crest and the buccal bone plate (H_C−B_) was evaluated to represent the atrophy of the buccal aspect of the alveolar crest. More pronounced bone resorption can be expected at the buccal aspect after tooth extraction, and the resulting ridge defect often includes a vertical component with a height discrepancy between the buccal and the lingual aspect of the ridge [13, 33]. When the H_C−B_ was higher, the alveolar ridge had a more sloped configuration and presented with a knife-edge morphology. In a canine histological study, Araujo et al. reported that the height discrepancy between the buccal and lingual alveolar bone plates was approximately 2 mm at 8 weeks after tooth extraction [34]. Because the discrepancy between the buccal and lingual bone heights could not be directly measured, CBCT scans were used in the current study for radiographic measurements, which made the level of the alveolar crest easier to determine than the most coronal point of the lingual plate. Among the included edentulous molar sites in the current study, 86/158 presented with > 2 mm of H_C−B_. These findings were similar to those of a previous study evaluating the bone dimensions of mandibular edentulous molar sites [30]. Braut et al. [30] measured the discrepancy in bone width between 4 mm apical to the alveolar crest and at the superior border of the inferior alveolar canal to evaluate the caudal divergence of the buccal and lingual plates, and the results showed that 65.3% of mandibular edentulous molar sites were knife-edge ridges.

The present study was the first to assess the relationship between the KMW and alveolar bone dimensions of edentulous molar sites. When the sites were grouped according to H_C−B_, the mean KMW of the sites with > 2 mm of the H_C−B_ was significantly lower than that of the sites with less than 2 mm of the H_C−B_ (*p* < 0.001). The correlation analysis also revealed that the KMW was negatively associated with the H_C−B_ (*p* < 0.001). The buccal bone plate underwent more resorption than the lingual bone. Therefore, the alveolar crest shifted lingually and presented with knife-edge ridges, which was more evident in molar regions [20, 21, 23, 34]. In the present study, the H_C−B_ was lower at maxillary molar sites, and the KMW was greater. Similarly, a previous study showed that the buccal MGJ changed minimally after 6 months of spontaneous healing at maxillary posterior sites [24]. As the buccal bone resorption continued over time, the MGJ shifted coronally, and shrinkage of the KM occurred, leading to a “V-shaped” alveolar ridge compared with the MGJ of neighboring teeth [35]. The present study showed that the KMW was narrower at edentulous sites with a high H_C−B_. The sloped configuration of the alveolar crest and limited ridge width affect the design of the implant location and the treatment plan, and additional bone augmentation procedures may be needed, during which the attempt at flap closure could result in a further decrease in the KMW [7, 36, 37]. Schiegnitz et al. [23] used implants with a sloped shoulder at sites with a sloped profile of the alveolar ridge, indicating that the KMW could be slightly increased. Thus, soft tissue augmentation should be performed at these sites before implantation if necessary, or specially designed implants should be chosen to adapt the configuration of the alveolar ridge to preserve more KMW for long-term peri-implant tissue health after implant treatment [7, 38].

The alveolar bone height was not significantly associated with the buccal KMW at edentulous molar sites. This lack of association could be due to more evident changes in the horizontal ridge dimensions following tooth loss [11, 39]. A recent systematic review indicated that the horizontal ridge reduction of molar postextraction sites was 3.61 mm, while the vertical resorption values of the buccal and lingual plates were 1.46 mm and 1.20 mm, respectively [39]. Considering the heterogeneity of alveolar bone height among individuals, it may be better to prospectively observe the changes in the ABH and KMW over time and explore the relationship between them.

Numerous factors can influence the degree of postextraction hard and soft tissue dimensional alterations. During the early weeks after tooth extraction, the process of bone remodeling was active, spontaneous soft tissue coverage after healing provided an increased amount of keratinized mucosa, and the soft tissue thickness exhibited a sevenfold spontaneous increase after healing at sites with thin bone wall phenotypes [26]. When the evaluation was performed after an average of 282 days of edentulism, the crestal soft tissue was significantly thicker at anterior sites than at posterior sites, while no significant associations were detected between the crestal soft tissue thickness and bone dimensions [40]. As time progresses, alveolar bone resorption is often accompanied by a decrease in the KMW and reduced vestibular depth with a lack of functional stimulus and a lack of vascular blood supply due to missing periodontal ligaments [5]. Although teeth suffering from severe periodontal disease already exhibit marked bone loss and gingival recession, the KMW of edentulous sites was comparable in the present study irrespective of the cause of tooth loss, which was consistent with the results of our previous study [32].

Techniques such as alveolar ridge preservation (ARP) were applied to maintain the alveolar ridge volume after tooth extraction [41, 42]. ARP has been demonstrated to preserve more KM than spontaneous healing, and ARP with flapless procedures can achieve better outcomes for preserving KM than flapped procedures [43, 44]. Further studies should evaluate the effect of ARP using different materials or wound closure techniques on preserving KM after tooth extraction to support the current findings.

The present study had several limitations. First, due to the retrospective design, the height discrepancy between the buccal and lingual bone plates could not be measured directly. Second, the curved contour of the alveolar ridge sometimes makes the most coronal point of the buccal bone plate indistinct and difficult to determine, which involves some uncertainty. To better account for this uncertainty, measurements were repeated, and examiners calibrated the data to improve the quality of the data collection. Third, the accuracy of the clinical measurement of the KMW at the edentulous ridge needs to be improved because the measurement is usually conducted on a curved surface. Evaluating the KMW along the soft tissue profile as a surface distance using oral scans or other novel devices may improve the accuracy of the assessment [45]. Fourth, because alveolar ridge resorption is an inevitable process following tooth loss, the effect of the time elapsed from extraction on the KMW should also be considered. While the patients included in the present study had missing molars for more than six months or even years, recall bias could be present if the time at which tooth extraction was performed was based on patient memories. Fifth, the pattern of bone resorption in the anterior region is different from that in the posterior region following tooth loss, the results of the current study should be limited to the molar region and interpreted cautiously. A prospectively designed study that includes an evaluation of the periodontal phenotype before tooth extraction and the dynamic postextraction changes in the KMW and bone dimensions would help to better understand the process, and a larger sample size may provide a more thorough analysis.

## Conclusions

Within the limitations of the current study, it can be concluded that the buccal KMW at edentulous molar sites is significantly associated with the height discrepancy between the alveolar crest and the buccal bone plate. Alveolar ridges presenting with a sloped configuration are more prone to a lower KMW. A prudent implant treatment design should be made for edentulous sites with a sloped configuration and narrow KMW, and soft tissue augmentation procedures should be performed before implantation if GBR is subsequently needed to achieve optimal, functional and esthetic rehabilitation after implant reconstruction.

## Data Availability

The datasets used and/or analyzed during the current study are available from the corresponding author on reasonable request.

## Abbreviations

KMW: Keratinized mucosa width
MGJ: Mucogingival junction
H_C−B_: Height discrepancy between the alveolar crest and the buccal bone plate
ABH: Alveolar bone height
CBCT: Cone-beam computed tomography
ICC: Intraclass correlation coefficient
GEEs: Generalized estimation equations
SD: Standard deviation
CI: Confidence interval
